# Modified protocol for enhanced recovery after surgery is beneficial for Chinese cancer patients undergoing pancreaticoduodenectomy

**DOI:** 10.18632/oncotarget.18092

**Published:** 2017-05-23

**Authors:** Xiaxing Deng, Xi Cheng, Zhen Huo, Yuan Shi, Zhijian Jin, Haoran Feng, Yue Wang, Chenlei Wen, Hao Qian, Ren Zhao, Weihua Qiu, Baiyong Shen, Chenghong Peng

**Affiliations:** ^1^ Department of Surgery, Ruijin Hospital, Shanghai Jiao Tong University School of Medicine, Shanghai 200025, China

**Keywords:** enhanced recovery after surgery, pancreatic cancer, pancreaticoduodenectomy, perioperative management

## Abstract

Radical surgical resection remains the only effective treatment for advanced pancreatic cancer. Effective protocols for recovery from post-operative complications that result in high rates of morbidity and mortality are therefore essential. The enhanced recovery after surgery (ERAS) protocol is an interdisciplinary multimodal concept based on modern anesthesia and analgesia combined with other fast rehabilitation parameters. It was first applied in the field of elective colorectal surgery, and eventually extended to several surgical diseases. In this study, we investigated the feasibility and safety of implementing the ERAS protocol in patients undergoing pancreaticoduodenectomy (PD). We randomly divided 159 patients who underwent PD into two groups who were managed using either ERAS or the conventional protocol. We observed that in those treated with the ERAS protocol several post-operative recovery factors were greatly improved, and there were no complications requiring readmission. We therefore propose that ERAS can improve post-operative recovery of PD patients and shorten the waiting time to chemotherapy, which may improve the overall survival of surgically treated pancreatic cancer patients.

## INTRODUCTION

Pancreatic cancer (PC) is one of the most lethal human malignancies that ranks fourth among the cancer-related deaths worldwide. 85% of patients are diagnosed in an advanced stage and this is reflected in a poor 5-year survival rate of only 5% [1, 2]. Radical surgical resection remains the only effective treatment for PC. Malignant tumors of the head and the periampullary region of the pancreas are surgically treated by pancreaticoduodenectomy. Pancreaticoduodenectomy is a high-risk procedure that was popularized by Whipple in 1935 [3] and has high perioperative morbidity and mortality. With recent advancements in both surgical and anesthetic techniques, the mortality rates have decreased to 5% and morbidity rates to 60% [4]. However, post-operative complications, such as pancreatic fistula, delayed gastric emptying and biliary complications result in prolonged hospitalization that average 14-28 days outside the USA [5]. The efficacy of traditional perioperative and postoperative care for patients undergoing pancreaticoduodenectomy has been questioned due to lack of evidence.

Recently, enhanced recovery after surgery protocol (ERAS) was initiated to reduce surgical stress and maintain patient homeostasis. ERAS was launched first in the field of elective colorectal surgery in the 1990s [6, 7] and has rapidly gained popularity because of its significant benefits and safety [8]. However, due to fewer case studies in pancreatic surgery its efficacy for pancreatic cancer patients is inconclusive. Therefore, we explored the feasibility and safety of implementing ERAS in patients undergoing pancreaticoduodenectomy.

## RESULTS

### Comparative evaluation of demographic and intra-operative variable parameters

The ERAS and the conventional group of patients demonstrated similar demographic and basic parameters such as age, gender and weight (Table 1). The perioperative period protocol compliance rate was similar in the ERAS group (90%) to the conventional group (95%)(P>0.05). The co-morbidity characteristics that included three major diseases, namely, diabetes, cardiac-cerebral vascular disease and respiratory disease were also similar for both the groups (*p*=1). Although the operating time in the ERAS group was slightly longer (288±50.31 min) than the conventional group (276±55.88 min), the difference was not statistically significant (*p*=0.72). The blood loss volume was also comparable in the two groups (353±145.25 ml for ERAS vs 411±133.26 ml for conventional; p=0.25). Further, intraoperative liquid infusion was significantly lower in the ERAS group due to restrictions (2400±522.12ml in ERAS *vs*. 3200±415.62ml in conventional; *p*=0.02).

**Table 1:** Demographic and intra-operative parameters of the 2 groups

Parameters	ERAS (n=76)	Conventional(n=83)	P
Age	54.5±12.7 (33-84)	51.3±15.0 (37-78)	0.55^a^
Sex(M/F)	46/30	46/37	0.46^b^
Co-morbidities	15	14	1^b^
Diabetes	6	8	0.74^b^
CCVD(cardiac-cerebral vascular disease)	7	4	0.35^b^
RD(respiratory disease)	3	2	0.63^b^
Preoperative total bilirubin(mmol/L)	51.5±1.8	44.2±3,2	0.32^a^
Perioperative albumin(g/L)	37.2±6.7	35.8±7.6	0.82^a^
ASA score			
≤II	54	64	0.62^b^
>II	22	19	0.31^b^
Jaudice	15	21	0.55^b^
Operation			
Operating time(min)	288±50.31	276±55.88	0.72^a^
Blood loss volume(ml)	353±145.25	411±133.26	0.25^a^
Intra-operative liquids(ml)	2400±522.12	3200±415.62	0.02^a^
TNM stage			
II	37	43	0.87^b^
III	39	40	0.96^b^
ECOG*			
≤1	72	76	0.76^b^
>2	4	7	0.54^b^
Surgical margin			
R0	61	58	0.83^b^
R1	15	25	0.14^b^

* ECOG: Eastern Cooperative Oncology Group.

^a^ Student's *t* test

^b^ Fisher exact test

### Comparative evaluation of post-operative courses

The ERAS group patients had a shorter stay in the ICU compared to conventional group (4.0±1.3 days *vs*. 4.2±2.1 days; *p*= 0.733). Further, the naso-gastric tube that was inserted right before the operation started was removed significantly earlier in the ERAS group than the conventional group (5.2±2.8 days *vs*. 8.4±3.5 days; *p*= 0.012, Table 2). Once the naso-gastric tube was removed, patients were allowed to have an oral liquid diet followed by a gradual return to oral solid diet (about 2 days after liquid diet). In case of discomfort or development of any other complications, the naso-gastric tube was reinserted. In our study, the ERAS group patients returned to the oral liquid diet earlier than the conventional group (5.4±1.6 days for ERAS *vs*. 9.6±2.5 days for conventional; *p*=0.007). Also the ERAS group patients defecated earlier (2.9±1.6 days) than the conventional group patients (3.7±2.2 days; *p*=0.041). This resulted in the drain tubes being removed significantly earlier in the ERAS group (10.2±3.1 days) than the conventional group (15.2±3.9 days; *p*=0.038).

**Table 2:** Post-operative parameters of the 2 groups

Parameters	ERAS (n=76)	Conventional (n=83)	P^a^
Naso-gastric tube removed (days)	5±3	8±4	0.012
Oral liquid diet (days)	5±2	10±3	0.007
Oral solid diet (days)	7±3	12±4	0.032
First defecation (days)	3±2	4±2	0.041
Drain tube removed (days)	10±3	15±4	0.038
Stay in ICU (days)	4±1	4±2	0.733
Post-operative hospital stay (days)	15±8	19±10	0.024

^a^ Student's *t* test

### Comparative evaluation of post-operative complications

Incidences of post-operative complications among the patients are documented in Table 3. Certain surgical complications like pulmonary complication, sepsis, wound infection and abdominal abscess were similar in the two groups. However, only 15 patients in the ERAS group had delayed gastric excretion (DGE) compared to 32 in the conventional group (*p*=0.02). Further, two patients in the ERAS group had pulmonary complications compared to four in the conventional group. Among the four conventional group patients, two had a MRSA bacterial infection in the ICU. Bleeding was noted in six ERAS group patients compared to four conventional group patients, although statistically it wasn't significant. An artificial orifice was placed outside the anterior abdomen wall with skin stitches to anchor the surgical drainage tubes. We observed that only one ERAS group patient showed skin orifice infection compared to six in the conventional group (*p*=0.012). The pancreatic fistula incidences were comparable in both groups (39 in ERAS *vs*. 36 in conventional; *p*=0.52). Only one ERAS group patient had pancreatitis compared to two in the conventional group. Of the four patients that had re-laparotomy, three belonged to the ERAS group and one belonged to the conventional group. Two of the four patients had to undergo re-laparotomy due to post-operative hemorrhage and one each due to biliary fistula and pancreatic fistula, respectively. There were no mortalities in the two groups of patients although one patient in each group had to be readmitted.

**Table 3:** Post-operative complications and mortalities of the 2 groups

Parameters	ERAS (n=76)	Conventional (n=83)	P^a^
Delayed gastric emptying	15	32	0.02
Bleeding	6	4	0.5
Pancreatic fistula	39	36	0.52
Grade A	8	6	
Grade B	10	6	
Grade C	21	24	
Biliary fistula	2	0	0.47
Wound infection	5	3	0.66
Pulmonary complication	2	4	0.1
Skin orifice infection	1	6	0.012
Abdominal abscess	0	0	/
Pancreatitis	2	1	1
Sepsis	1	0	1
Re-laparotomy	3	1	0.6
Mortality	0	0	/
Readmission (in 30 days)	1	1	1

^a^ Fisher exact test

## DISCUSSION

Despite advances in diagnostic and therapeutic management, prognosis of PC still remains poor. Radical surgical resection of the tumor remains the only effective treatment for PC with a 5-year survival rate of only 5% [9]. The unique position and the special function of the pancreas coupled with its high post-operative morbidity rate renders pancreatic surgery as one of the most difficult surgeries. In comparison, advancements in modern surgery, anesthesiology and perioperative care have made pancreaticoduodenectomy much safer with acceptable morbidity and mortality. In prestigious medical facilities, the mortality rates are as low as 5%, although maintaining high morbidity (30% to 60%) [10–13]. Further, postoperative complications such as pancreatic fistula, hemorrhage and biliary complications still require high-level perioperative care leading to prolonged hospitalization [14, 15]. Although traditional perioperative and postoperative care exists for patients undergoing pancreaticoduodenectomy, their efficacy has been questioned due to lack of evidence.

ERAS is an interdisciplinary multimodal concept that is based on modern anesthesia and analgesia combined with other rehabilitation parameters. Ever since the ERAS concept was introduced by Kehlet [9], it has been applied to several surgical diseases including radical prostatectomy, cardiac surgery, total knee replacement, cesarean section and other procedures for children and the elderly [16, 17]. ERAS consists of a number of evidence-based interventions that are associated with improved outcomes following major surgery. The three main targets for ERAS include physiological stress reduction, vital function support and reduction of postoperative morbidity. ERAS rehabilitation has made great progress in the past ten years with several studies on colonic resection demonstrating its effectiveness [18, 19]. However, ERAS has not yet been established in pancreatic surgery. Therefore, in this study, we evaluated the benefits of ERAS particularly regarding morbidity, mortality, post-operative complications and rehabilitation and assessed if it reduced the length of hospitalization.

Several studies have indicated that routine naso-gastric tube is unnecessary in elective abdominal surgery since it can lead to increased pulmonary complications and prolonged nausea and vomiting [20]. However, due to the complicated reconstruction of pancreatojejunostomy and cholecystojejunostomy, we implemented naso-gastric tube for gastrointestinal decompression and to decrease anastomotic leakage. Further, we removed the naso-gastric tube earlier in the ERAS group that allowed return to oral diet earlier. This also helped decrease any related complications as only two ERAS patients reported pulmonary complications compared to four in the conventional group. Moreover, there was no increase in the incidence of anastomotic leakage. Therefore, our use of naso-gastric tube for a short period was beneficial to ERAS group patients.

Nearly 20-30% of patients undergoing pancreatic surgery demonstrate delayed gastric excretion (DGE) [21]. The cause of post-operative DGE is still controversial and probably is due to pyloric, antral and/or duodenal ischemia, [abdominal complications, imbalance of hormonal and neuronal factors and obstructions related to digestive tract reconstruction [23–25]. We started oral liquid and solid diet earlier in the ERAS group (15.2±7.5 days) compared to the conventional group (19.1±9.7 days; *p*=0.024) and observed fewer instances of DGE (15 in ERAS *vs*. 32 in conventional; p=0.02). More importantly, we did not observe enhanced incidences of the pancreatic fistula as a result of starting oral liquid and solid diet earlier (51.3% in ERAS *vs*. 43.4% in conventional, *p*=0.152). Therefore, our data suggest that ERAS promotes gastrointestinal peristalsis and reduces DGE [22, 23].

Pancreatic fistula is the most common and challenging complication following pancreatoduodenectomy that can trigger lethal delayed massive hemorrhage and septicemia [24, 25]. Risk factors for pancreatic fistula were: (1) general factors like advanced age, jaundice, and malnutrition; (2) procedure-related factors such as intra-operative blood loss, prolonged operative time, resection type, soft pancreatic parenchyma, small pancreatic duct and anastomotic technique [26]. The surgical technique is the most important factor contributing towards pancreatic fistula. Also, most deaths directly resulted from pancreatic fistula complication followed by a delayed massive hemorrhage. Traditionally, longtime drainage tube flushing was used to treat pancreatic fistula. However, certain surgeons believe that long time routine drainage increases the incidence of intra-abdominal abscess, wound infections, exacerbated abdominal pain, reduced lung function, eroded hollow viscera and peripancreatic vessels and therefore prolongs hospitalization [27]. In this study, inspite of significant shorter drainage placement time in the ERAS group (10.2±3.1 for ERAS *vs*. 15.2±3.9 for conventional, *p*=0.041), pancreatic fistula instances were comparable between the ERAS and the conventional groups (39 *vs*. 36; *p*=0.152, with a rate of 51.3% *vs*. 43.4%). Further, the drainage tube related complications like pulmonary complications and skin orifice infections were lower in the ERAS group. Therefore, we observed improvements not only in physiological stress relief but also in regard to hospitalization in ERAS group. Most importantly, there were no differences in the mortality and 30 day readmission rates between the two groups. Altogether, ERAS decreased the discomfort and complications resulting from surgical drainage.

The surgical burden from pancreaticoduodenectomy, the post-operative complications and the slow recovery of digestive function or physical strength influenced the prognosis remarkably, especially for patients that rehabilitated slowly and could not tolerate adjuvant chemotherapy initially (23.7 days for ERAS *vs*. 38.9 days for conventional, p<0.05). In conclusion, although our ERAS protocols were based on colonic surgery, the results in this study demonstrated that the ERAS protocol significantly relieved physiological stress and accelerated recovery thereby reducing the length of hospitalization of pancreatic cancer patients. It also does not increase the incidence of complications such as pancreatic fistula, post-operative hemorrhage and mortality. Thus, our results confirmed that ERAS was beneficial and safe for patients undergoing pancreaticoduodenectomy.

## MATERIALS AND METHODS

### Patient recruitment and characteristics

In August 2012, ERAS protocol was introduced in the Department of surgery, Ruijin hospital, Shanghai Jiao Tong University School of Medicine. For the current study, 159 patients were recruited from August 2012 to August 2014. We obtained informed consent from the patients regarding the experimental procedures that were approved by the Ethics Committee of Ruijin Hospital, Shanghai Jiao Tong University School of Medicine. The study was conducted in accordance with the international Helsinki declaration. The data on these patients, such as age, gender, smoking history, BMI, biochemical profiles, coagulation profiles, serum tumor marker level, past pancreatic disease history, and preoperative ERCP were prospectively collected in this study. All patients were aware of the potential risks and complications of the proposed treatment scheme, and treatment compliance was measured using indirect methods including self-reported compliance, evidence of expected side effects, and so on. The 159 patients were randomly into two groups with 76 patients enrolled into the ERAS protocol and 83 patients managed according to the conventional perioperative care protocol following elective pancreaticoduodenectomy. The simple randomization was performed via a computer-generated randomization chart immediately after hospitalized. The patients were full aware of perioperative protocol and equally divided into the ERAS group or the Convention group.

### Pancreaticoduodenectomy procedure

All 159 patients received classical pancreaticoduodenectomy performed by the same panel of Hepatic-Biliary-Pancreatic specialists. The pylorus-preserving PD (PPPD) procedure was performed with end-to-side pancreaticojejunostomy, end-to-side hepaticojejunostomy and retrocolic duodenojejunostomy. All anastomoses were hand-sewn. One silicon drain was placed near the pancreatic intestinal anastomosis. All cases underwent a complete CT scan examination before the surgery and were evaluated for the possibility of R0 resection based on the exclusion criteria (widespread tumor metastasis, adhesion with nearby organs, vessels invasion and widespread peritoneal metastasis). Preoperative diagnoses demonstrated that, 122 cases had tumors of the head of pancreas and 37 cases had malignant tumor of the periampullary region.

### Enhanced recovery after surgery protocol

The ERAS protocol was established and modified based on colonic surgery (Table 4). Briefly, the day before the surgery, the patients avoided oral solid food intake after 8 pm and light meal was allowed until 10 pm. Clear liquids could be taken until 2 hour before the procedure All patients received infection prophylaxis 30min before surgery with a single dose of 1.5g Cefuroxim. Antibiotics were administered repeatedly if surgery lasted longer than 3h. Moreover, low-molecular weight heparin was given to prevent weight-adapted thrombosis and pancreatic secretion inhibitor octreotide (0.2mg) was administered thrice. During the operation, the fluid rate was maintained at 2 ml·kg^-1^·h^-1^ to avoid any fluid overload. After surgery, patients were transferred to either ICU or the normal ward according to ASA (American Society of Anesthesiologists) status standards. Post-operative pain medication (Flurbiprofen 300mg, tramadol 60 mg in 100-ml saline solution) was administered through an elastomeric pump (1.5-2.0 ml/h) until the third post-operative day. Naso-gastric tubes were usually removed on the second or third day post-operatively if the volume was less than 200ml. Oral intake of clear liquids was resumed as soon as possible after extubation and oral intake was increased gradually from liquid to mashed to light and low-fat normal diet. When the patients returned to the normal ward, they were mobilized at least 4 times a day. Urinary catheters were removed on day 3. The abdominal drainage tubes were removed in 7-10 days if the drainage volume was less than 200ml with no apparent abnormal fluid drainage. Pancreatic and biliary fistulas were monitored as well. Further details of the ERAS protocol are summarized in Table 4.

**Table 4:** Parameters for patients on the enhanced recovery after surgery program

**Day before surgery**	Normal oral nutrition until 10 pm
	No pre-anaesthetic medication
	Preoperative information given to patient, including daily milestones
**Day of surgery**	Elastomeric analgesia pump:(flurbiprofen 300mg, tramadol 60 mg in 100-ml saline solution)
	Warm i.v. fluids, and upper and lower air-warming device
	Avoidance of excessive i.v.fluid
	First night in ICU (intensive care unit)
**Day 1-2**	Patient sent back to surgical ward
	Removal of naso-gastric tube if<200ml
	Patient mobilized at least 4 times a day
**Day 2**	Continue mobilization minimum 4 times per day
	Sip of warm water at rate≤30ml/h
	Metoclopramide os to prevent nausea and vomiting
**Day 3**	Urinary catheters removed
	Stop elastomeric pump
	Clear oral liquid
	Enhanced mobilization
**Day 4**	Soft solid diet
**Day 5**	Dietary increase on daily basis
	Medical oncology and radiaation oncology consults(if appropriate)
**Day 7-10**	Removal of drainage tubes if no pancreatic/biliary fistula and <200ml
**Discharge**	Absence of fever for more than 48h
**Day 8-11**	Able to take solid food
	Passage of normal stools
	Adequate mobilization
	Acceptance of discharge by the patient

### Conventional perioperative care protocol

The conventional perioperative parameters included routine perioperative bowel preparation with regular oral antibiotics and no oral intake for 12 hours before surgery. The naso-gastric tube was kept in place until day 7 after surgery with no scheduled early mobilization. The oral liquid intake was resumed from day 7 and a stepwise oral intake recovery was allowed with only water for the first two days followed by resumption of liquid diet during the next 4 days. Later, mashed hard food intake was allowed.

No significant differences were observed regarding demography profile or pre-operative parameters between the two groups. Comparison of rest of the parameters between the two groups including intra-operative parameters, post-operative recovery parameters, perioperative morbidity and mortality are listed in Tables 2 and 3.

### Post-operative outcome measures and discharge criteria

The outcome measures included postoperative complications, length of post-operative hospital stay and 90-day mortality rates. We also evaluated complications like pancreatic fistula, biliary fistula, delayed gastric emptying, intra-abdominal abscess, post-pancreatectomy hemorrhage, wound infection and acute pancreatitis. We defined pancreatic fistula as persisting secretions of the drainage fluid on or after post-operative days 7-10 with three fold greater amylase content than the upper normal serum value. Biliary fistula was defined as fluid with a high level of bilirubin (>3 times the bilirubin serum level) secreted for more than 5 days. Delayed gastric emptying was defined as continuous drainage via the gastric tube (more than 500ml/day) for more than 5 days after surgery with recurrent vomiting in combination with swelling of the gastrojejunostomy / duodenojejunostomy and dilatation of the stomach at radiological contrast examination. The definition of abdominal abscess was collection of fluid of at least 5cm in diameter as diagnosed by CT scan or ultrasound accompanied by fever and leukocytosis. Acute pancreatitis was defined as a three or more fold increase in normal plasma amylase or lipase values 48h following surgery in the context of an appropriate clinical picture.

The discharge criteria were: (1) absence of fever for more than 48 hours; (2) no symptomatic pancreatic or biliary fistula; (3) ability to take semi-solid food; (4) ability to pass normal stool; (5) adequate mobilization and (6) acceptance of discharge by the patient.

### Statistical analysis

Statistical analysis was performed with SPSS (version 17.0, SPSS Inc. Chicago, III, USA). Fisher exact test or Student's *t* test was used to evaluate significant differences. A p value < 0.05 was considered statistically significant. Data were represented by mean±SD or median±SD.
